# The use of antibiotics to improve phage detection and enumeration by the double-layer agar technique

**DOI:** 10.1186/1471-2180-9-148

**Published:** 2009-07-23

**Authors:** Sílvio B Santos, Carla M Carvalho, Sanna Sillankorva, Ana Nicolau, Eugénio C Ferreira, Joana Azeredo

**Affiliations:** 1 Institute for Biotechnology and Bioengineering, Centre of Biological Engineering, Universidade do Minho, Campus de Gualtar, 4700-057 Braga, Portugal

## Abstract

**Background:**

The Double-Layer Agar (DLA) technique is extensively used in phage research to enumerate and identify phages and to isolate mutants and new phages. Many phages form large and well-defined plaques that are easily observed so that they can be enumerated when plated by the DLA technique. However, some give rise to small and turbid plaques that are very difficult to detect and count. To overcome these problems, some authors have suggested the use of dyes to improve the contrast between the plaques and the turbid host lawns. It has been reported that some antibiotics stimulate bacteria to produce phages, resulting in an increase in final titer. Thus, antibiotics might contribute to increasing plaque size in solid media.

**Results:**

Antibiotics with different mechanisms of action were tested for their ability to enhance plaque morphology without suppressing phage development. Some antibiotics increased the phage plaque surface by up to 50-fold.

**Conclusion:**

This work presents a modification of the DLA technique that can be used routinely in the laboratory, leading to a more accurate enumeration of phages that would be difficult or even impossible otherwise.

## Background

Bacteriophages (phages) are viruses that specifically infect bacteria. They can be found in almost all ecosystems and it is estimated that approximately 10^31 ^phages exist globally (10^8 ^phage species predicted), making them the most prominent biological system on earth [1-5]. Despite these enormous numbers it is estimated that less than 1% of all phage species have been detected by the plaque assay because of undersampling, which is often attributed to the use of classical bacteriophage propagation procedures [4,5].

The ability of a phage to lyse its host bacterium, producing a plaque within a bacterial lawn, led to the discovery of phages in 1915 by Frederick W. Twort and is the basis of the classic plaque assay, the double-layer agar (DLA) technique, which has been used ever since [6-8] to identify and enumerate phages and isolate mutants. In recent years, interest in phages has increased not only because of their potential use as alternatives to antibiotics (phage therapy) but also because of their applications in many other fields (phage display, immunology, microbial genetics, diagnostics, vaccine development, biosensors, etc.). Isolation, detection or/and enumeration of phages are essential in all these applications, and the increasing interest in phages has broadened the use of the DLA technique. As a consequence, the efficiency of this method has several implications in different areas of biology [9-11].

While many phages form plaques that are sufficiently large and well-defined to be detected and enumerated easily by the classical DLA technique, some give rise to small and turbid plaques that are difficult to detect and count accurately. In these cases, the classical plaque assay can be rather unsatisfactory and sometimes highly unreliable [4,12-14].

Various approaches have been proposed to enhance plaque morphology and hence the ease and accuracy of plate counts. The addition of dyes that bind specifically to cells in the bacterial lawn is the most common approach. The dyes most frequently used are tetrazolium salts (2,3,5-triphenyltetrazolium chloride, 2,5-diphenyl-3 [alpha-naphthyl]-tetrazolium chloride). Unfortunately, Hurst *et al*. [15] have reported that this dye results in titer suppression in more than 70% of phages tested [11-17]. A combination of ferric ammonium citrate and sodium thiosulfate (FACST) has also been employed to enhance plaque visualization. However, this only works with bacterial strains that produce hydrogen sulphide, which is a major limitation. In addition, plaque counts have to be made within 12 h of plating because the black lawns tend to fade rapidly [13,18].

Antibiotics have been found to influence phage growth. Price and Krueger independently reported that in general more phage formed in the presence than the absence of penicillin [19-22]. More recently, Hadas *et al*. [23] and Maiques *et al*. [24] observed that beta-lactam antibiotics stimulated phage development in *Escherichia coli *and *Staphylococcus aureus*, and Comeau *et al*. [25] observed that sub-lethal concentrations of aztreonam and cefixime stimulated phage production by a uropathogenic *E. coli *strain. These few reports imply that at least some antibiotics, under certain conditions, have the ability to stimulate bacteria to produce phage, increasing their final concentration. This effect may thus be used to increase phage plaque size, improving the efficacy of the DLA technique.

In this work we studied the conditions under which antibiotics can increase plaque size leading to the isolation, identification and more accurate enumeration of phages that would be difficult or even impossible otherwise.

## Methods

### Media

The medium used in this work was LB broth, Miller (Sigma-Aldrich Inc., St. Louis MO – USA), prepared according to the manufacturer's instructions. It was used for bacterial growth in the suspension in which the bacterial lawn was prepared. For use in the DLA method, this same medium was supplemented with agar (Applichem, Darmstadt – Germany) at final concentrations of 1.2% and 0.6% for bottom and top agar respectively. When stated, glycerol (Applichem, Darmstadt – Germany) was added at the required concentration to the top, bottom or both layers before sterilization.

### Antibiotics

Ampicillin, penicillin G, kanamycin, rifampicin and tetracycline hydrochloride were purchased from Sigma-Aldrich Inc. (St. Louis MO – USA) while cefotaxime was obtained from Labesfal-Laboratórios de Almiro SA (Amadora – Portugal). They were dissolved in distilled water and filter-sterilized using a 0.22 μm PES syringe filter from Tpp-Techno Plastic Products AG (Trasadingen – Switzerland) prior to addition to the media.

### Phages

All phages used in this work are virulent and are listed in Table 1 along with their sizes and hosts. The phages were isolated from sewage (purified by several isolation of single plaques) and represent the three families in the order Caudovirales, which include 96% of all observed phages [16]. The *Pseudomonas fluorescens *phage phi IBB-PF7A was already described by Sillankorva *et al *[26]. Phage dimensions were determined by Dr. Hans-W. Ackermann (Université Laval, Quebec, Canada – personal communication).

**Table 1:** Phages used.

PHAGE	FAMILY	DIMENSIONS (nm)	HOST
**phi PVP-SE1**	*Myoviridae*	Tail:120 × 18; head: 84	*Salmonella enterica *Enteritidis

**phi PVP-SE2**	*Siphoviridae*	Tail:125 × 8; head: 57	*Salmonella enterica *Enteritidis

**phi IBB-PF7A**	*Podoviridae*	Tail:13 × 8; head: 63	*Pseudomonas fluorescens*

**phi IBB-SL58B**	*Podoviridae*	Tail:13 × 9; head: 64	*Staphylococcus lentus*

### Determination of phage titer

The titer of each phage, expressed as plaque forming units (pfu), was determined using the DLA technique as described by Sambrook and Russel [27]. Briefly, 100 μl of a dilution of the phage sample was added to 100 μl of a bacterial suspension grown overnight at 37°C, 120 rpm. This solution was added to 4 ml top agar, gently homogenized, and poured into a 90 mm petri dish (Plastiques-Gosselin, Borre – France) previously prepared with 10 ml bottom agar. The plates were gently swirled, dried for 10 min at room temperature and then inverted and incubated at 37°C overnight.

To test the effects of antibiotics on plaque size, the corresponding antibiotic was added at the concentration desired to the bottom, top or both agar layers after sterilization of the medium. Glycerol was added to the top, bottom or both layers before sterilization.

### Phage plaque size

Pictures of the plates were taken with a Hewlett-Packard Scanjet 3300C scanner, using a black background to avoid distortion and to allow equal light exposure and contrast conditions in all photographs. The photographs were not adjusted for brightness, contrast or colour. In order to obtain accurate dimensions, the diameter and area of the plaques were automatically determined from photographs at 4-fold magnification using the computer image analysis program Sigma Scan Pro, version 5.0.0 of SPSS Inc (Chicago – USA). Each value is the average of up to 20 plaque measurements.

### Microscopic observation of bacterial cells

Bacterial cells were grown for 7 h in LB with or without glycerol and supplemented with an antibiotic (0.5 mg/l ampicillin, 0.06 mg/l cefotaxime or 1.5 mg/l tetracycline). The bacterial suspension was then diluted 1:20 in saline (0.9% NaCl) and washed twice in saline (centrifuged at 5000 rcf for 10 min). A 20 μl drop was placed on a glass slide and left to air-dry. The sample was then counterstained with 30 μl (10 μg/ml) 4',6'-diamidino-2-phenylindole (DAPI) from Sigma-Aldrich Inc. (St. Louis MO – USA) and incubated for 10 min in the dark. Excess DAPI was removed and the sample was allowed to air-dry, mounted with non-fluorescent immersion oil (Merck, Darmstadt – Germany) and covered with a coverslip. Finally, the cells were visualized under an Olympus BX51 epifluorescence microscope (Olympus Portugal SA, Lisbon – Portugal) equipped with a filter sensitive to DAPI fluorescence.

### Statistical analysis

To test for differences among groups the data obtained for phage titer determination (counts of up to 30 plates) were subjected to statistical analysis using one-way ANOVA (confidence level 99.9%), version 5.0.0 of SPSS Inc (Chicago – USA).

## Results

**Figure 1:**
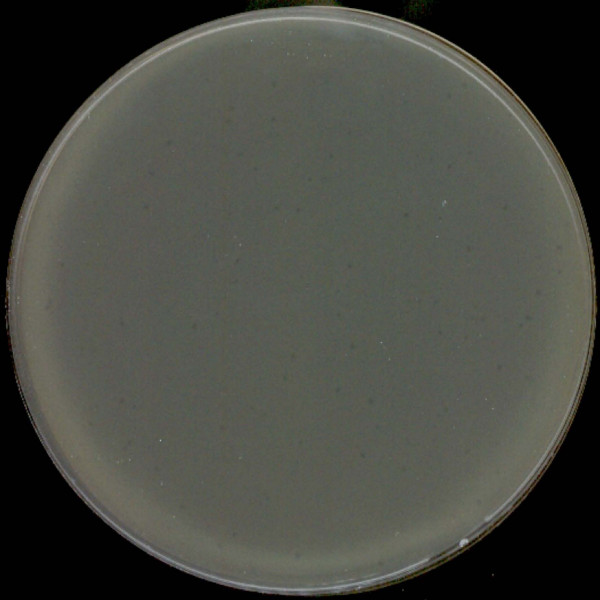
**Plaques of phi PVP-SE1 obtained by classical DLA technique**.

**Figure 2:**
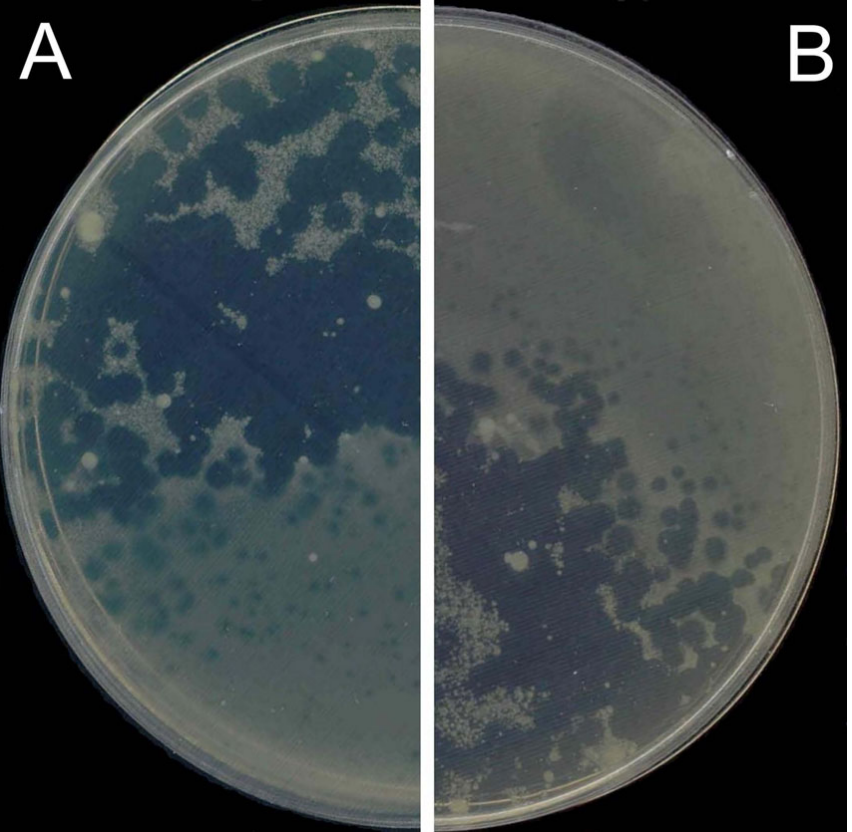
**Heterogeneous phi PVP-SE1 plaque increase with 2 mg/l ampicillin added to the top layer**. A and B – different plates with 2 mg/l ampicillin.

As part of the European Project Phagevet-P, a *Salmonella *phage (phi PVP-SE1), characterised by a broad lytic spectrum, was isolated. Unfortunately, according to the DLA technique, this phage produces very small and turbid plaques that are very difficult to detect and enumerate (Figure 1). The development of a method for improving the visualization of phage plaques was essential. We therefore studied the ability of different antibiotics and glycerol to enhance plaque size. When the DLA technique is modified by the addition of antibiotics (and glycerol) it is referred to as PAMA (Plaque Assay Modified with Antibiotic). Antibiotics were incorporated at different concentrations in the top agar layer. Only four of them increased plaque size: penicillin G, ampicillin, cefotaxime and tetracycline. With this approach a notable increase in plaque size was observed, but plaque size and lawn distribution were very heterogeneous (Figure 2). To overcome this problem we tested the addition of the antibiotic to the bottom agar layer only and to both layers. Plates more homogenous in plaque size and bacterial lawn distribution were obtained only when the antibiotics were added to both layers. No further experiments with penicillin G were carried out once the concentration needed to obtain a plaque size increase exceeded 20 mg/l (much higher than the other antibiotics).

**Figure 3:**
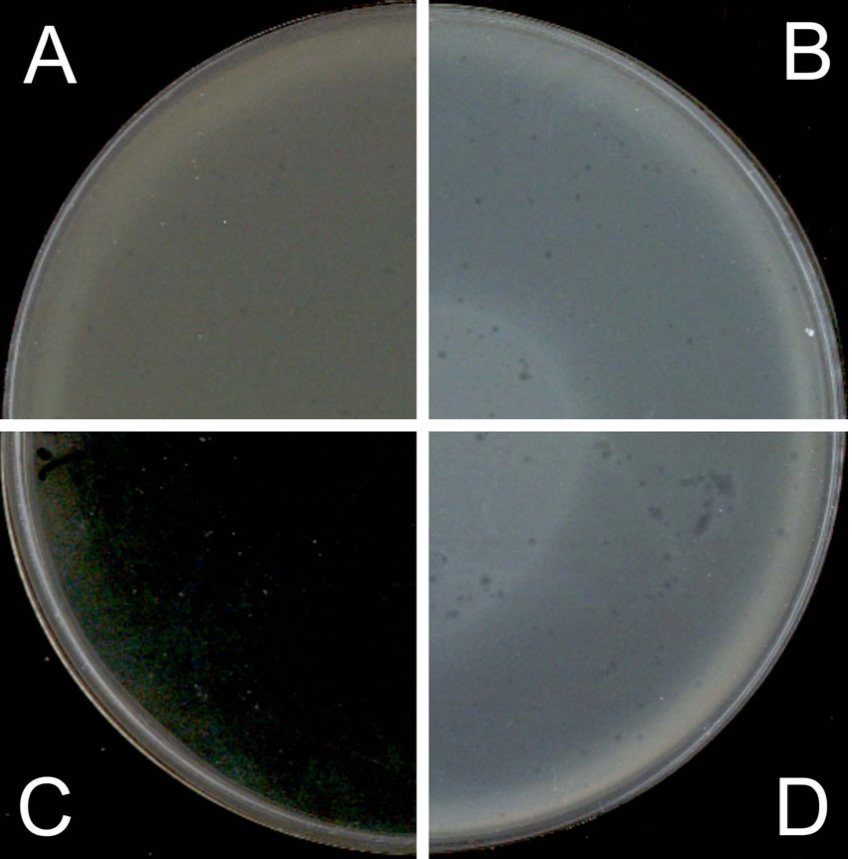
**Determination of optimal glycerol concentration using plaques of phi PVP-SE1**. A – without glycerol (0%); B – with 5% glycerol; C – with 20% glycerol; D – with 10% glycerol.

The effect of glycerol at three final concentrations (5%, 10% and 20%) in both layers (without antibiotics) was tested and compared with a control containing no glycerol or antibiotic (Figure 3). The best improvement in plaque observations was achieved with 5% glycerol, where we obtained a small increase in plaque size and a very good increase in contrast. Thus, further experiments were carried out using this concentration.

**Figure 4:**
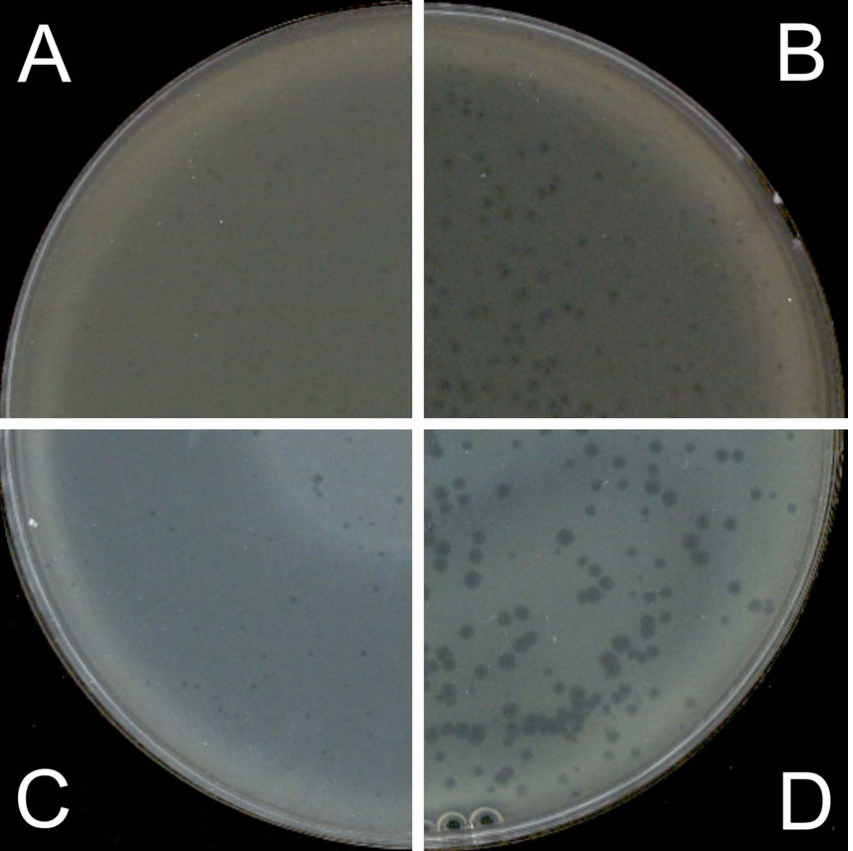
**Influence of 5% glycerol in the top layer on phi PVP-SE1 phage plaques**. A – classical DLA; B – PAMA with 0.2 mg/l cefotaxime; C – as in A but with 5% glycerol; D – as in B but with 5% glycerol.

The combination of glycerol and antibiotics produced larger plaques and a dramatic increase in contrast compared with the use of antibiotics alone (Figure 4). In this way, glycerol appears to act synergistically with antibiotics in improving plaque observations.

**Figure 5:**
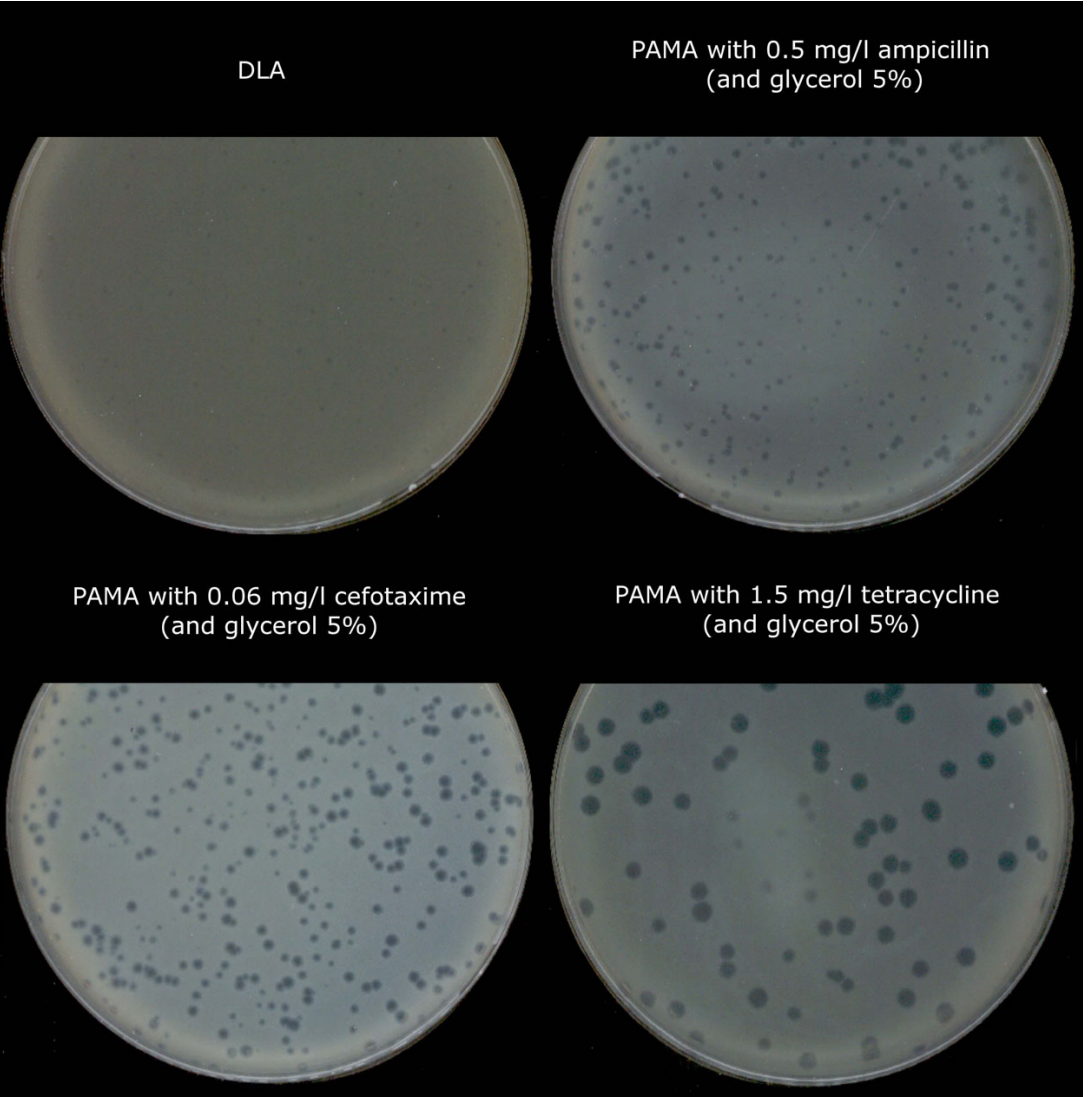
**Optimized conditions for improvement of phi PVP-SE1 plaques**.

**Figure 6:**
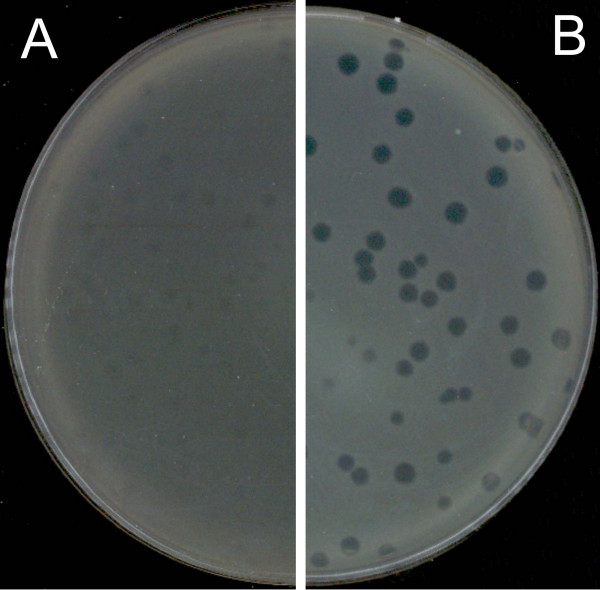
**Influence of glycerol in phage phi PVP-SE1 plaque improvement**. A – with tetracycline alone at 1.5 mg/l; B – with 1.5 mg/l tetracycline and 5% glycerol.

The optimum antibiotic concentration should be the highest possible to produce the maximum increase in plaque size but not so high that it inhibits bacterial lawn formation. Therefore, the effects of different antibiotic concentrations in both layers were analyzed, and the following optimal concentrations were determined: 0.5 mg/l ampicillin, 0.06 mg/l cefotaxime and 1.5 mg/l tetracycline (Figure 5). Comparing these antibiotic concentrations with and without glycerol (Figure 6) we concluded that glycerol critically improves plaque observation, especially for tetracycline, for which both the plaque size and contrast were increased. Tetracycline was the antibiotic that induced the highest increment of phage plaque size and contrast (Table 2).

**Table 2:** Comparison of phage phi PVP-SE1 plaque diameter with DLA and with PAMA using different antibiotics.

	DLA	AMP [0.5]	CEF [0.06]	TET [1.5]
**PLAQUE DIAMETER (mm)**	0.47 ± 0.167	1.49 ± 0.433	1.91 ± 0.439	3.43 ± 0.398

**AREA INCREASE**	1	10	17	53

Values of plaque diameters are expressed in mm±standard deviation and area increase as the ratio between the average values of each method and DLA. DLA: classical Double-Layer Agar technique; AMP [0.5]: PAMA with 0.5 mg/l ampicillin; CEF [0.06]: PAMA with 0.06 mg/l cefotaxime; TET [1.5]: PAMA with 1.5 mg/l tetracycline.

**Figure 7:**
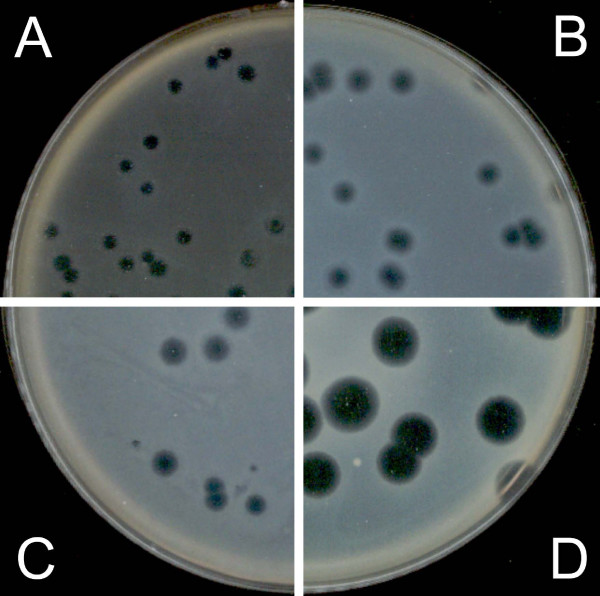
**Influence of PAMA on phi PVP-SE2 phage plaques**. A – Classical DLA; B – PAMA with 0.5 mg/l ampicillin and 5% glycerol; C – PAMA with 0.06 mg/l cefotaxime and 5% glycerol; D – PAMA with 1.5 mg/l tetracycline and 5% glycerol.

**Figure 8:**
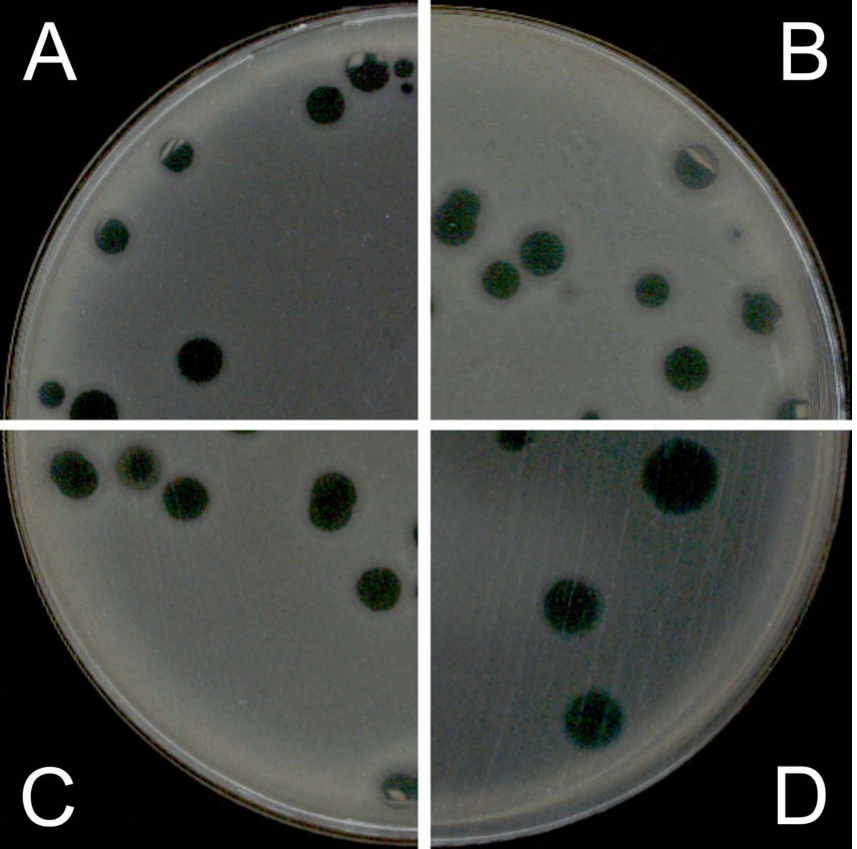
**Influence of PAMA on phi IBB-PF7A phage plaques**. A – Classical DLA; B – PAMA with 0.5 mg/l ampicillin and 5% glycerol; C – PAMA with 0.06 mg/l cefotaxime and 5% glycerol; D – PAMA with 1.5 mg/l tetracycline and 5% glycerol.

**Figure 9:**
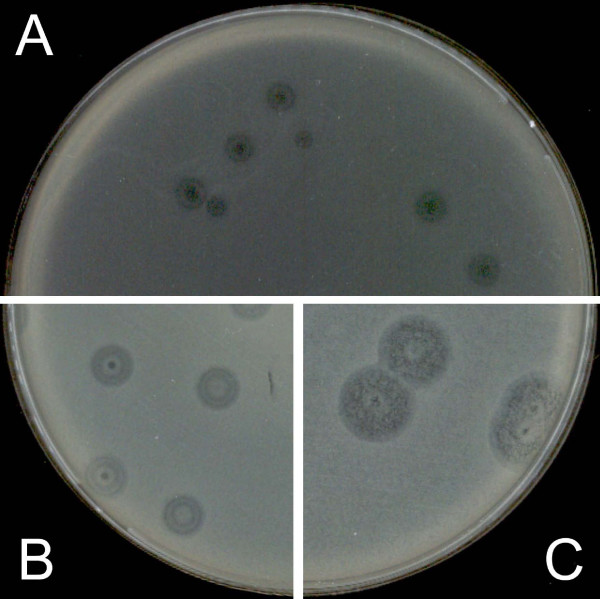
**Influence of PAMA on phi IBB-SL58B phage plaques**. A – Classical DLA; B – PAMA with 0.5 mg/l ampicillin and 5% glycerol; C – PAMA with 100 mg/l ampicillin and 5% glycerol.

These optimized antibiotic concentrations plus glycerol (5%) were applied to three other phage-host systems to assess their ability to increase phage plaque. With phage phi PVP-SE2 only a slight increase in plaques was observed when cefotaxime and ampicillin were used, while the addition of tetracycline produced an enormous increase in phage plaque size (Figure 7). There was no significant effect on the plaquing behaviour of *Pseudomonas fluorescens *phage phi IBB-PF7A (Figure 8). In the case of *Staphylococcus *phage phi IBB-SL58B, ampicillin at 50–100 mg/l resulted in a very significant increase in plaque size (Figure 9).

It was important to ensure that the glycerol and antibiotics caused no diminution of plaque numbers. We addressed this issue by comparing the phage titers determined in the classical DLA procedure and the newly-developed PAMA method (Table 3). The average phage titer (in pfu) was statistically significantly higher when antibiotics were used (PAMA) (p < 0.001 for each antibiotic), justifying rejection of the null hypothesis (that are no differences between groups with and without PAMA) with a confidence of 99.9%. The higher phage titer value could be due to the release of prophages from the host bacterium as a result of induction, as described for Mitomycin C. However, this hypothesis is false since the host bacteria stressed with antibiotics released no prophage. A better explanation is erroneous determination of the phage titer by the traditional DLA method. The fact that the phage plaques are very small rendered accurate counting almost impossible. In order to assess the suitability of this method for phage enumeration, another experiment was carried out using phage phi PVP-SE2, which forms large, well-defined plaques. This eliminates the risk of miscounting plaques that are difficult or impossible to observe with the naked eye in the classical DLA technique (Table 3). The experiment showed that the differences in phage titers determined by DLA and PAMA were not statistically significant (p > 0.01).

**Table 3:** Comparison of phage titer determinations with DLA and with PAMA using different antibiotics

	DLA	AMP [0.5]	CEF [0.06]	TET [1.5]
	**phi PVP-SE1**			

**PFUs (average ± SD)**	14 ± 5	55 ± 10	53 ± 11	58 ± 10

**SD %**	38	19	21	17

	**phi PVP-SE2**			

**PFUs (average ± SD)**	54 ± 4	54 ± 5	48 ± 11	51 ± 3

**SD %**	8	8	23	6

DLA: classical Double-Layer Agar technique; AMP [0.5]: PAMA with 0.5 mg/l ampicillin; CEF [0.06]: PAMA with 0.06 mg/l cefotaxime; TET [1.5]: PAMA with 1.5 mg/l tetracycline.

**Figure 10:**
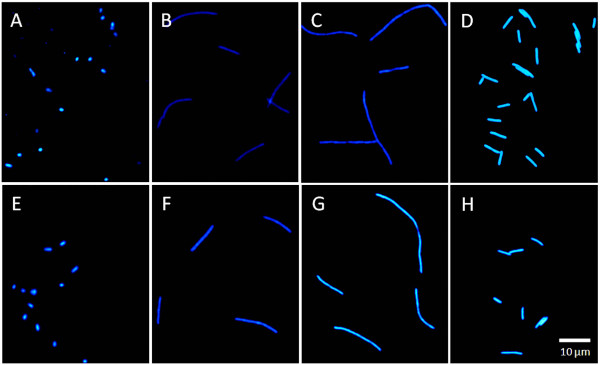
**Microscopic observation of phage phi PVP-SE1 host (S1400/94)**. A – LB only; B – LB with 0.5 mg/l ampicillin; C – LB with 0.06 mg/l cefotaxime; D – LB with 1.5 mg/l tetracycline; E – LB and 5% glycerol; F – LB with 0.5 mg/l ampicillin and 5% lglycerol; G – LB with 0.06 mg/l cefotaxime and 5% l glycerol; H – LB with 1.5 mg/l tetracycline and 5% glycerol.

Microscopic observation of the phage phi PVP-SE1 host cells (Figure 10) showed that when these cells were stressed with antibiotics, especially cefotaxime (Figure 10C, G) or ampicillin (Figure 10B, F), they filamented extensively. Tetracycline produced a smaller increase in cell size (Figure 10D, H). The addition of glycerol (Figure 10E–H) induced no observable alteration in cell morphology compared to cells grown in unmodified LB (Figure 10A–D).

## Discussion

Plaque development has been the subject of several recent reviews [28-32]. Plaque size seems to be directly proportional to burst size, phage adsorption constant and the diffusion of phages in the medium and inversely proportional to the latent period, each factor contributing differently [25,28,29]. A decrease in the latent period and an increase in burst size has been observed in the presence of antibiotics [19-25]. The enhancement of phage production by antibiotics is reported to be due to bacterial filamentation [25]. Krueger *et al*. observed that penicillin-treated *S. aureus *produced filaments three times the diameter of normal bacteria [19] and enhanced phage development. Hadas *et al*. also found that bacterial cells exposed to this antibiotic were 4-fold larger and the yield of phage production was enhanced by an equal amount.

Burst size also increases in parallel with DNA content but not with DNA concentration [23]. Thus, it seems that cell size rather than metabolic rate is a major influence on phage development in the presence of antibiotics. Further experiments showed that the rate of phage production is proportional to the amount per cell of the protein synthesizing system (PSS) at the time of infection and is not limited by cell size or DNA composition [23,33]. In fact, larger faster-growing cells contain proportionally more PSS leading to higher phage production. Thus, cell size does not play a primary role in increasing phage production but has an indirect effect by increasing PSS. As a result, because some antibiotics trigger the SOS system, the bacterial cells will divide poorly, increasing their size and resulting in cell filamentation, which in turn will increase their PSS content, thus enabling an increase in phage production. From this we can conclude that any stimuli that increase PSS content will increase phage production and plaque size, and such stimuli may act indirectly by filamentation or inducing the SOS response. This seems to explain why glycine stimulates plaque formation, as in the work presented by Lillehaug. This amino acid has been shown to weaken the bacterial cell wall, which induces the SOS response and consequently increases the PSS content. This fact has remained hitherto unexplained [10,23,33]. As a consequence, any substance or condition (e.g. agitation or temperature) that directly or indirectly stimulates an increase of PSS is able to increase phage production and thus plaque size.

The adsorption rate is also influenced by antibiotics: it is directly proportional to cellular surface area and therefore increases when cells are subjected to some antibiotics, as observed by Hadas *et al*. (1997) [23,33].

The only antibiotics previously reported to increase phage production belong to the beta-lactam and quinolone groups, which are known to induce the SOS system. Indeed, this effect was not observed with other classes of antibiotics [19-25]. In the present work and for the first time, an effect similar to that of beta-lactams is reported with tetracycline. Curiously, this antibiotic induced larger plaques than beta-lactams. In the light of the foregoing discussion, this may be expected since it is well established that tetracycline can cause cell elongation and filamentation, so it is potentially able to increase phage production [34-36]. However, in the light of the results obtained, filamentation (or cell size elongation) seems not to be the only determinant of plaque size increase. In fact we observed that tetracycline induced the greatest increase in plaque size, but cells subjected to it were smaller than those incubated with the other antibiotics tested. Indeed, we found no correlation between plaque size and cell size.

An unexpected observation in this work was the conspicuous effect of glycerol in increasing phage plaque size and contrast. Glycerol produced a huge improvement in plaque observations when tetracycline was used. It allowed plaques to be observed that had very little contrast and were difficult to observe when tetracycline alone was used. This difficulty in observing the plaques obtained with tetracycline and no glycerol may explain why the effect of tetracycline, and even of other classes of antibiotics, has not been observed previously. We conclude that glycerol plays a critical role in improving plaque observation. Glycerol may increase phage diffusion in the medium resulting in enhanced plaque size. Since it is a nonfermentative carbon source for these bacteria its presence will result in increased biomass or delay the onset of stationary phase. A plaque is unlikely to increase in size as the lawn cells enter late log growth stage [10,37-39].

All in all, the influence of antibiotics on burst size, latent period and adsorption rate and the influence of glycerol on the diffusivity of phages in the medium and on bacterial growth seem to act together leading to a great increase in plaque size. Moreover, it was demonstrated here that antibiotics not only have the ability to increase phage plaques, they also do not suppress bacteriophage development at subminimal inhibitory concentrations (sub-MICs).

In addition, the present results allow us to conclude that the new method (PAMA) can be applied to both Gram-negative and Gram-positive bacteria with lytic phages. The phages used represent the three families in the order Caudovirales, which include 96% of all observed phages [16]. Obviously, the antibiotic to be used in the PAMA, as well its concentration, have to be optimized for each bacterial host.

## Conclusion

It is well known that some phages in the classical DLA technique produce plaques that are difficult or impossible to observe with the naked eye, leading to erroneous phage enumeration. In this work it was observed that low concentrations of certain antibiotics facilitate an increase in plaque size and improve plaque visibility without suppressing phage development. For the first time it was observed that tetracycline can improve phage plaques. It was also observed that glycerol critically enhances these improvements. In addition, it was found that induction of cell filamentation produces an increase in plaque size although there was no direct correlation between cell size and plaque size.

In conclusion, the work presented in this paper is a simple modification of the DLA technique that produces an increase in phage plaques, improving their visibility, and can be used for virulent viruses of both Gram-negative and Gram-positive bacteria. As a consequence it allows phages to be enumerated easily and accurately (manually or automatically), which would otherwise be very difficult or impossible. This method might also enable new phages to be detected and counted that have previously been overlooked because they cannot form readily visible plaques.

Furthermore, this work has contributed to an explanation of why antibiotics are able to improve plaque size and increase phage production.

## Authors' contributions

SBS designed, planned and performed the experiments, analyzed the data and made the statistical analysis, drafted, articulated and wrote the manuscript. CC participated in the design and execution of experiments. SS provided the phages phi IBB-PF7A and phi IBB-SL58B. AN participated in the design of experiments. EF and JA supervised and participated in the conception of the study and contributed with reagents, materials and statistical tools. All authors read and approved the final manuscript.
